# Human Prion disease with a T188K mutation in Chinese: a case report

**DOI:** 10.1186/1757-1626-2-7820

**Published:** 2009-05-29

**Authors:** Qi Shi, Chen Gao, Wei Zhou, Bao-Yun Zhang, Chan Tian, Jian-Ming Chen, Hui-Ying Jiang, Jun Han, Xiao-Ping Dong

**Affiliations:** State Key Laboratory for Infectious Disease Prevention and Control, National Institute for Viral Disease Control and Prevention, Chinese Center for Disease Control and PreventionYing-Xin Rd 100, Beijing 100052People's Republic of China

## Abstract

Inherited Prion diseases are characterized by mutations in the *PRNP* gene predispose to disease by causing the expression of abnormal PrP protein. We report a 58-year-old Chinese female with mutation in codon 188 (T188K) of the *PRNP* gene, while the codon 129 was a methionine homozygous genotype. The patient displayed 4-year long slowly progressive sleeping disturbance and rapid exacerbation of neurological status after other neurological manifestations appeared. Cerebral spinal fluid 14-3-3 protein was positive.

## Introduction

Transmissible spongiform encephalopathies (TSEs), or prion diseases, are a group of neurodegenerative diseases of central nerve system (CNS). The pathological agent of TSEs, termed as PrP^Sc^, is believed to be an abnormal isoform of a cellular prion protein, PrP^C^. Roughly 85% human TSEs occur sporadically, named sporadic Creutzfeldt-Jacob disease (sCJD) [1]. About 5-15% cases are related with a range of mutations in the prion protein gene (*PRNP*) on chromosome 20 by causing the expression of abnormal PrP protein, including familiar CJD (fCJD), Gerstmann-Sträussler-Scheinker syndrome (GSS) and fatal familial insomnia (FFI) [2]. The rests are believed to be infected by exogenous prion agents, i.e. iatrogenic CJD that is infected by human CJD agents through medical services and variant CJD that is infected by consuming the food contaminated by bovine spongiform encephalopathy (BSE) agent.

Two types of mutations in *PRNP*, including point mutations and octapeptide repeats insertions, have been identified to be associated or directly linked to human TSEs. In this report, we describe a Chinese patient who presents lethargy and progressive dementia. A novel nucleotide mutation was detected in one *PRNP* allele, resulting in an exchange of the amino acid of codon 188 (T188K).

## Case presentation

A Chinese female, 58-year-old farmer, complained to suffer from excessive daytime sleepiness for four years. Her family members described that she slept 2 to 3 hours at beginning, and 5 to 6 hours later at daytime and sometimes fell asleep during working in the field. Two months before admission to hospital, she had persistent dizziness, tiredness, lethargy and complained to see objects shaking in front of her. She was described to be fractious and splenetic, often quarreled with her family members. One month before admission, the strength of her right limbs became weakened and often dropped things, and her body inclined to right obviously during walking. She was reluctant to speak and developed memory disturbance. Half month ago, the strength of her left limbs became weakened too and she needed support with others when she walked. During hospitalization, the patient was bedridden and had intellectual deterioration. Myoclonus and tremor were observed at the state of waking or sleeping. Neurological examination revealed slightly hyperreflexia and bilateral Babinski sign. EEG displayed sharp waves in bilateral frontal lobe. Brain CT scan showed no abnormality. Biochemistry tests of CSF (including protein, glucose and cell count) were almost normal, except that the chloride concentration (134 mM) was slightly higher than the normal value (range between 118-128 mM). Retrospective investigation of her pedigree did not reveal similar neurological disorders. The patient died in hometown after 10 days discharged from the hospital.

**Figure 1.**
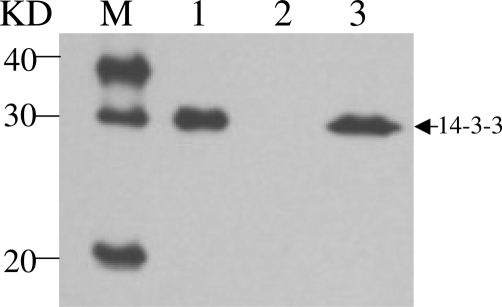
Western blot of 14-3-3 in CSF. Lane 1: 10% sheep brain homogenates as positive control; Lane 2: previously confirmed 14-3-3 negative human CSF sample as negative control; Lane 3: the CSF sample of the patient. M represents the protein markers. The position of 14-3-3 is indicated by arrow.

Protein 14-3-3 in CSF was performed as follows. Briefly, 20 µl CSF sample was separated by 12% SDS-PAGE and electronically transformed onto nitrocellulose membrane. Blots were incubated in 1:1000 diluted 14-3-3 polyclonal antibodies (Santa Cruz, CA, USA) and further incubated in 1:5000 diluted HRP-conjugated goat anti-Rabbit IgG. Immunoreactive bands were visualized by ECL method (PerkinElmer, Germany). Clearly positive signal migrating at 30 KD was detected in the patient's CSF (Figure 1).

**Figure 2.**
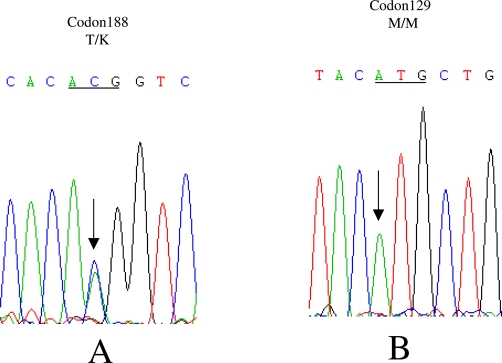
Sequencing gels showing a C to A heterozygous transition (T188K) at codon 188 in one *PRNP* allele **(A)** and the homozygosis for methionine at codon 129 of *PRNP* from the patient **(B)**.

Genomic DNA was extracted from peripheral blood leukocytes by using Qiagen's DNA purification kit according to the manufacturer's instructions. The *PRNP* open reading frame was amplified by polymerase chain reaction (PCR) and the genotype at codon 129 of *PRNP* was determined by digestion with the restriction endonuclease *NspI*. Analysis of *PRNP* sequences was performed by direct sequencing in a MacBAC sequencer (Pharmacia, USA). A missense mutation (C to A) at the position of nt 563 in one *PRNP* allele was identified, leading to change from threonine to lysine at codon 188 (Figure 2A). No other nucleotide exchange was found in the rest of the *PRNP* sequence. The codon 129 genotype was methionine homozygous (Figure 2B) confirmed by *NspI* digestion and directly sequencing of the amplified product. Because of unwilling donation of blood sample, the distribution of T188K mutation in her family was not applicable.

## Discussion

Here we report the first patient with T188K inherited prion disease in Chinese. It also represents the second reported CJD case with T188K worldwide, [3]. The Chinese patient shows some similar clinical features as the German one, e.g. rapidly progressive dementia, failure of cognitive abilities, myoclonus, visual disturbances and CSF 14-3-3 positive. The onset ages of the German and Chinese patients are quite same. Both two patients have negative family histories of dementia or related neurological symptoms. Interestingly, the Chinese patient has 4 year-long slowly excessive daytime sleepiness, however, after appearance of salient neurological manifestation, her general and neurological status declines rapidly (roughly three months). Unfortunately, brain postmortem is not available, thus, whether presence of typical CJD neuropathological changes, especially thalamus impairment that is usually associated with sleep disturbance, is not addressable.

In addition to T188K mutation, two other mutations, T188A and T188R, have been described to be linked with genetic CJD [4,5]. The T188A patient is an 82-year old Australian woman, who suffered from sCJD-like symptoms and died 4 months later. The T188R case is identified from a large *PRNP* gene survey of suspected prion disease referred to German CJD surveillance unit. The exact information of this case is not accessible. Expression of mutant PrP T188K and PrP T188R in mammalian cells illustrates that these PrP mutants possess PrP^Sc^-like properties, i.e. enhanced PK-resistance and detergent insolubility [6]. It seems that codon 188 in human *PRNP* is a hypervariable region. Because of limited cases, whether and how the diversity of the mutations at codon 188 influences clinical phenotype remains still unclear.

## List of abbreviations

BSE: Bovine spongiform encephalopathy
CNS: Central nerve system
CSF: Cerebral spinal fluid
fCJD: familiar CJD
FFI: Fatal familial insomnia
GSS: Gerstmann-Sträussler-Scheinker syndrome
sCJD: Sporadic Creutzfeldt-Jacob disease
TSEs: Transmissible spongiform encephalopathies
